# Editorial Notes

**Published:** 1882-07

**Authors:** 


EDITORIAL NOTES.

Notice to Graduates of Bellevue Hospital Medical College.—A second decennial revision of the catalogue of alumni of this college is being prepared for publication, and we are requested to ask that all graduates send their present address, at once, on a postal card, to the Historian of the Alumni Association, Bellevue Hospital Medical College, New York, N. Y.

